# Loss of Insulin Receptor in Osteoprogenitor Cells Impairs Structural Strength of Bone

**DOI:** 10.1155/2014/703589

**Published:** 2014-05-18

**Authors:** Kathryn Thrailkill, R. Clay Bunn, Charles Lumpkin, Elizabeth Wahl, Gael Cockrell, Lindsey Morris, C. Ronald Kahn, John Fowlkes, Jeffry S. Nyman

**Affiliations:** ^1^Department of Pediatrics, University of Arkansas for Medical Sciences, Little Rock, AR, USA; ^2^Arkansas Children's Hospital, 1 Children's Way, Slot 512-6, Little Rock, AR 72202, USA; ^3^Arkansas Children's Hospital Research Institute, Little Rock, AR, USA; ^4^Division of Diabetes, Endocrinology & Metabolism, Department of Medicine, Vanderbilt University School of Medicine, Nashville, TN, USA; ^5^Joslin Diabetes Center and Harvard Medical School, Boston, MA, USA; ^6^VA Tennessee Valley Health Care System, Vanderbilt University Medical Center, Nashville, TN, USA; ^7^Department of Orthopaedic Surgery & Rehabilitation, Vanderbilt University Medical Center, Nashville, TN, USA; ^8^Center for Bone Biology, Vanderbilt University Medical Center, Nashville, TN, USA

## Abstract

Type 1 diabetes mellitus (T1D) is associated with decreased bone mineral density, a deficit in bone structure, and subsequently an increased risk of fragility fracture. These clinical observations, paralleled by animal models of T1D, suggest that the insulinopenia of T1D has a deleterious effect on bone. To further examine the action of insulin signaling on bone development, we generated mice with an osteoprogenitor-selective (osterix-Cre) ablation of the insulin receptor (IR), designated OIRKO. OIRKO mice exhibited an 80% decrease in IR in osteoblasts. Prenatal elimination of IR did not affect fetal survival or gross morphology. However, loss of IR in mouse osteoblasts resulted in a postnatal growth-constricted phenotype. By 10–12 weeks of age, femurs of OIRKO mice were more slender, with a thinner diaphyseal cortex and, consequently, a decrease in whole bone strength when subjected to bending. In male mice alone, decreased metaphyseal trabecular bone, with thinner and more rodlike trabeculae, was also observed. OIRKO mice did not, however, exhibit abnormal glucose tolerance. The skeletal phenotype of the OIRKO mouse appeared more severe than that of previously reported bone-specific IR knockdown models, and confirms that insulin receptor expression in osteoblasts is critically important for proper bone development and maintenance of structural integrity.

## 1. Introduction

Skeletal health is compromised in persons with type 1 diabetes mellitus (T1D), as characterized by decreased bone mineral density (BMD), a deficit in bone structure, and subsequently an increased risk of fragility fractures [1, 2]. Indeed, T1D has been listed among the top 10 factors associated with the highest risk of fracture [3]. Because T1D is characterized in humans and rodents as a state of insulin deficiency due to destruction of pancreatic beta cells (either autoimmune-mediated or chemically induced), a role for insulinopenia in the pathogenesis of osteoporosis and increased fracture risk, now categorized as diabetic bone disease, has been postulated [4].

Insulin has been demonstrated* in vitro* and* in vivo* to be an osteogenic hormone. Primary osteoblasts and osteoblast-like cells in culture bind insulin and respond to insulin treatment [5–7], as is evidenced by increased cell proliferation rates [8, 9], enhanced collagen synthesis [6, 10–12], secretion of bone formation markers [13], and increased uptake of glucose [14, 15].* In vivo*, systemic insulin therapy can prevent or reverse skeletal deficits which occur in rodent models of T1D, including improving bone strength and biomechanical integrity [16–19]. Even* local *insulin delivery to fracture sites in diabetic rodents can ameliorate poor fracture healing which occurs in T1D [20]. Taken together, these data suggest that insulin may play a critical role in normal osteoblastogenesis and that conditions of insulin deficiency may lead to abnormal skeletogenesis.

A direct link between insulin signaling and bone formation* in vivo* is supported by transgenic mouse models. Knockdown of the insulin receptor (IR) in osteoblasts results in altered bone formation [21] and in abnormal trabecular architecture [22]. These studies support a role for insulin in mature osteoblasts; Ferron et al. [21] report a 60% knockdown of the* Insr* using the* Col1a1-Cre* mouse, while Fulzele et al. [22] have eliminated the* Insr *in mature osteoblasts using the* osteocalcin- (OC-) Cre* construct.

To examine further the specific action of insulin signaling on bone, we generated mice lacking the insulin receptor in osteoprogenitor cells. Unlike previous reports that deleted IR in mature osteoblasts, [21, 22] we assessed the effect of IR deletion at the early stages of osteoblast differentiation (osterix) on the architectural, structural, and biomechanical properties of bone. We hypothesized that the ablation of insulin signaling prenatally in cells of the osteoblast lineage via a loss of IR would impair the fracture resistance of mouse bones, independent of body size.

## 2. Materials and Study Design

### 2.1. Ethics Statement

This research protocol was approved by the Institutional Animal Care and Use Committee of the University of Arkansas for Medical Sciences.

### 2.2. OIRKO Mice

Our experimental objective was to generate mice lacking the insulin receptor in osteoblast lineage cells considered as precursors to previously reported models (i.e.,* Col1a1-Cre *or* osteocalcin- (OC-) Cre*). Therefore, to generate mice with an osteoprogenitor-selective ablation of the insulin receptor (OIRKO), we first crossed mice that were homozygous for a floxed insulin receptor (IR) allele in which* loxP* sites flank exon 4 of the IR gene (designated IR^*lox/lox*^) [23] with heterozygous* osterix- *(*Osx-) Cre* transgenic mice (B6.Cg-Tg(Sp7-tTA,tetO-EGFP/cre)1Amc/J; The Jackson Laboratory, Bar Harbor, ME). F1 progeny were crossed to produce IR^*lox/lox*^/*Cre* ^+/−^(OIRKO) and IR^*lox/lox*^/*Cre* ^−/−^ mice which were then bred to produce experimental animals. The IR^*lox/lox*^/*Cre* ^−/−^ mouse (IR flox) was designated as the control for this study, in keeping with previous reports [21, 22].* Cre* transgene expression is repressed when mice receive chow containing doxycycline (Diet S3888, Bio-Serv, Frenchtown, NJ). Upon switching mice to a regular chow diet (Diet 8640, Harlan, Indianapolis, IN) the* Cre* transgene is expressed in the osteoblast lineage, resulting in disruption of the floxed IR alleles in the osteoblast lineage. In this study, pregnant females were switched to regular chow approximately 1 week prior to birth of experimental pups. Nursing dams were maintained on regular chow and experimental mice were weaned to a regular chow diet. Genotyping was performed using published procedures [23].

**Figure 1:**
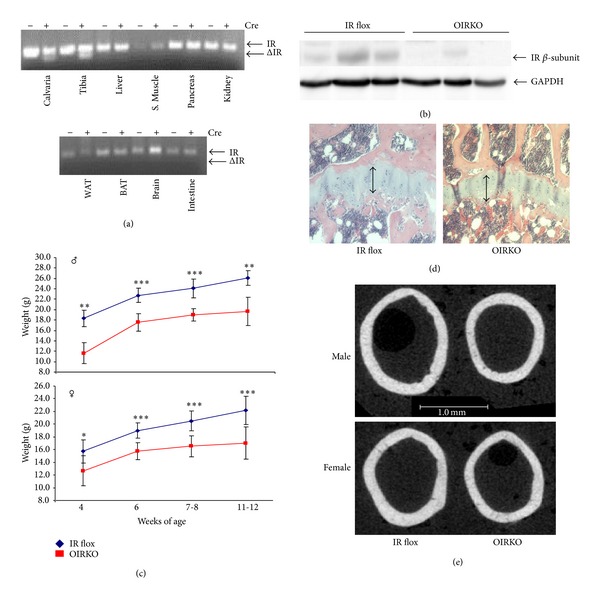
OIRKO mice are smaller in size, with slighter bones. PCR of the IR locus revealed that recombination of floxed IR alleles occurs only in bone ((a), ΔIR; calvaria, tibia). OIRKO mice exhibited an ~80% decrease in IR in whole bone by Western blot (*n* = 3 IR flox, 3 OIRKO tibiae, (b)). Loss of the insulin receptor in osteoblasts reduced the size of male and female mice. Postnatal growth curves for male ((c), top) and female ((c), bottom) IR flox and OIRKO mice are shown (*P < 0.05, **P < 0.01, and ***P < 0.001). Disruption in the growth plate was not apparent (a representative H & E stain is shown, (d)). *μ*CT images of the femur midshaft demonstrated that femurs from OIRKO mice are narrower than from comparably aged IR flox mice (e).

Postnatal growth, as assessed by weight gain, was monitored in both male (*n* = 15; IR flox (6) + OIRKO (9)) and female (*n* = 15; IR flox (6) + OIRKO (9)) mice at 4, 6, 8, and 10–12 weeks of age (Figure 1). Detailed metabolic and skeletal assessments were then completed.

### 2.3. Metabolic Assessment

Between 10-11 weeks of age in males and 12-13 weeks of age in females, a 150-minute glucose tolerance test (GTT) was performed. Following an overnight fast, blood glucose measurements were obtained from all animals before intraperitoneal injection of a 2 g/kg body weight glucose bolus. Blood glucose levels were then measured at 15, 30, 45, 60, and 150 minutes following the glucose bolus. In males only, insulin levels were also measured at 0, 15, 30, 45, 60, and 150 minutes. Blood glucose was measured via glucometer (OneTouch Ultra2 Blood Glucose Monitoring System, Lifescan, Inc., Milpitas, CA; average intra-assay coefficient of variation of 1.7% across a range of 40–300 mg/dL target glucose concentrations). Serum insulin levels were measured using a mouse insulin ultrasensitive ELISA (80-INSMSU-E01; ALPCO Immunoassays, Salem NH; sensitivity, 0.115 ng/mL, intra-assay coefficient of variation, 6.3%, inter-assay coefficient of variation, 10.61%). Glucose (males and females) and insulin (males only) area-under-curve (AUC), an integrated measure of glucose tolerance, was determined using GraphPad PRISM software (version 5.02). Serum total osteocalcin (OC) was measured using the MILLIPLEX MAP Mouse Osteocalcin-Single Plex assay (MBN-41 K-10C, Millipore Corp., Billerica MA; sensitivity, 8.7 pg/mL, intra-assay coefficient of variation, 11.2%, inter-assay coefficient of variation, 7.5%). Serum amino-terminal propeptide of type 1 procollagen (PINP) was measured using the Rat/Mouse PINP EIA (AC-33F1, Immunodiagnostics Systems, Ltd., Fountain Hills, AZ; sensitivity, 0.7 ng/mL). Serum c-terminal telopeptide of type 1 collagen, a marker of bone resorption, was measured using the RatLAPs ELISA (AC-06F1, Immunodiagnostics Systems, Inc., Scottsdale, AZ; sensitivity, 1.7 ng/mL, intra-assay coefficient of variation, 5.8%, inter-assay coefficient of variation, 10.4%).

### 2.4. Skeletal Assessment

After euthanasia (males: ~10-11 weeks; females: ~12-13 weeks), the left tibia and femur were harvested from IR flox (6 male and 6 female) and OIRKO (9 male and 9 female) littermates and stored in phosphate buffered saline (PBS) at −20°C until assessment. H & E staining of the tibial growth plate was performed as previously described [24]. Femur length was measured using calipers. Femurs were immersed in PBS at room temperature and the long axis of the bone was aligned with the scanning axis of the *μ*CT40 (Scanco Medical, Brüttisellen, Switzerland). All scans were acquired with an isotropic voxel size of 12 *μ*m using the same energy settings (70 kVp/0.114 mA), acquisition parameters (integration time of 300 ms with 1000 projections per full rotation), and calibration to a hydroxyapatite (HA) phantom with a beam hardening correction from Scanco Medical, as we have previously described [25]. The regions of interest (ROI) included the trabecular bone of the metaphysis (0.24–1.40 mm above the growth plate) and the cortical bone of the midshaft (1.19 mm in length spanning the midpoint between the condyles and femoral neck). Each ROI had a unique threshold (406 mgHA/cm^3^ and 774 mgHA/cm^3^ for trabecular and cortical bone, resp.) and Gaussian noise filter (sigma of 0.2 with support of 2 for trabecular bone and sigma of 0.8 with support of 2 for cortical bone) that were used for all scans. Standard evaluation scripts from the manufacturer were used to determine the architectural and structural properties of trabecular and cortical bone, respectively (Table 1). Moreover, in the calculations of tissue mineral density (TMD), partial volume effects were suppressed by peeling the first voxel from all surfaces following segmentation of the bone. The slenderness of the femur was calculated as the caliper-derived length of the bone divided by the *μ*CT-derived total cross-sectional area of the midshaft (i.e., average cross-sectional area including medullary canal).

### 2.5. Biochemical/Molecular Testing

Tissues were harvested from animals and immediately frozen in liquid nitrogen. Approximately 20 milligrams of tissue was digested at 58°C overnight in 200 *μ*L of buffer (50 mM Tris-HCl, pH 8.0, 0.5% Triton X-100) containing Proteinase K (1 mg/mL). One microliter of digested tissue was used in PCR reactions. Recombination of the floxed insulin receptor allele was evaluated by the polymerase chain reaction using the following primer pairs that amplify exons 3–5: 5′-GATGTGCACCCCATGTCTG-3′, 5′-CTGAATAGCTGAGACCACAG-3′, and 5′-ACGCCTACACATCACATGC-3′. PCR reactions were hot starting at 95°C for 15 minutes followed by 35 cycles of 94°C for 45 seconds, 60°C for 1 minute, and 72°C for 1 minute, with a final extension step of 72°C for 5 minutes. All reactions were performed on an MJ Research PTC-200 thermal cycler. Western blots were performed with antibodies directed against insulin receptor, beta subunit (Santa Cruz Biotechnology, Dallas, TX, catalog sc-711, 1 : 200), and GAPDH (EMD Millipore, Billerica, MA, catalog MAB374, 1 : 10,000) as described [26].

### 2.6. Biomechanical Testing

To determine the differences in strength between OIRKO and IR flox mice, a preload (1 N) held each hydrated femur in place on the lower support points of a three-point bending fixture with the anterior side down (i.e., bending about the medial-lateral plane). The span between the lower supports was 8 mm, and the load rate was 3.0 mm/min. Then, forces from a 100 N load cell (Honeywell, Morristown, NJ) and displacements from the LVDT (Dynamight 8841, Instron, Canton, OH) were recorded at 50 Hz during a monotonic load-to-failure test. Measured differences in biomechanical properties included stiffness and the peak force endured by the bone. Material properties of modulus and strength of the cortex were also estimated using standard beam theory [27]. The *μ*CT scans provided the moment of inertia and the distance between the neutral axis of bending and the outermost point in the anterior-posterior direction (*C*_min⁡_).

To ascertain if any skeletal differences in the OIRKO mice could be attributed to the presence of the* Cre* transgene alone, *μ*CT and three-point bending were also carried out in 8-week-old male mice that were* Cre* ^−/−^ or* Cre* ^+/−^ (Supplemental Table  1 in Supplementary Material available online at http://dx.doi.org/10.1155/2014/703589) on a wild-type background. (These mice were generated from breeding of IR^+/+^ */Osx*-*Cre* ^−/−^ and IR^+/+^ */Osx*-*Cre* ^+/−^ animals.)

### 2.7. Statistical Analysis

Statistically significant differences between groups (i.e., IR flox versus OIRKO) within each gender were detected using the unpaired Student's *t*-test. Data are reported as the mean ± the standard deviation (SD) unless otherwise specified, and differences were considered statistically significant at *P* < 0.05.

## 3. Results 

Prenatal elimination of the IR did not appear to affect fetal survival; OIRKO mice and IR flox control mice were born with the expected Mendelian frequencies. PCR of the IR locus indicated that recombination of floxed IR alleles (ΔIR) occurred only in bone (Figure 1(a); calvaria, tibia). Assessments of the efficiency and specificity of the IR knockout revealed an ~80% decrease (range: 44–97%) in IR in whole bone by Western blot (Figure 1(b)). Both male and female OIRKO mice appeared grossly normal; however, both male and female OIRKO mice displayed a postnatal growth constriction, evident by significant reductions in weight gain (Figure 1(c)) and by shorter femur length (Table 1). This occurred despite normally appearing growth plates (Figure 1(d)). Micro-CT images of the femur midshaft also demonstrated that femurs from OIRKO mice were narrower than those from IR flox mice (Figure 1(e)).

**Figure 2:**
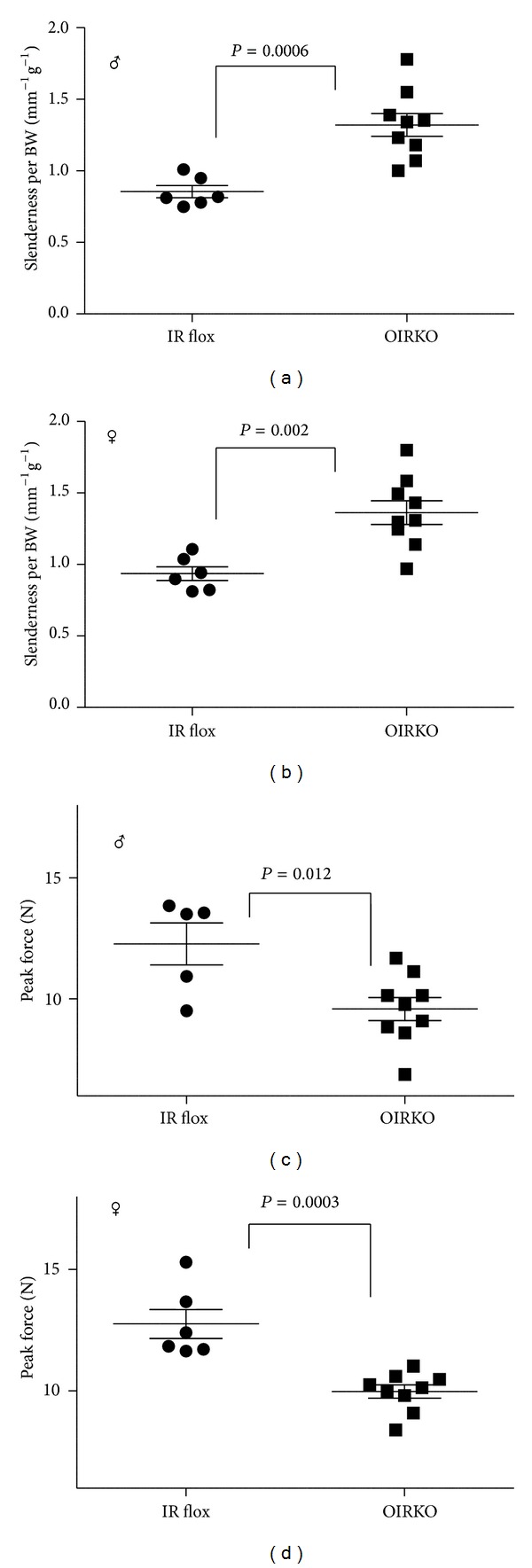
OIRKO femurs are weaker and more slender. Loss of IR in osteoblasts caused the bone to be more slender (ratio of length to total cross-sectional area) relative to body weight (Length/Tt.AR per BW; (a), male; (b), female). Loss of IR in osteoblasts also caused a decrease in the structural strength of the femur in bending (peak force; (c), male; (d), female). Data is displayed as a scatter-plot, with mean and SEM indicated.

Not surprising given their overall size reduction, OIRKO mice demonstrated a striking skeletal phenotype, but in a notably gender-specific manner. For male mice, the loss of IR in osteoprogenitors resulted in a lower trabecular bone volume fraction (BV/TV) with thinner (Tb.Th), more rodlike trabeculae in the femur metaphysis (Table 1). In contrast, for female OIRKO mice, trabecular bone was largely unaffected, with less rodlike trabeculae. In the diaphyseal compartment, however, the* cortices* of femurs from* both* male and female OIRKO mice were thinner and exhibited lower structural parameters (e.g., bone cross-sectional area or Ct.Ar) compared with the cortices from IR flox mice. With a smaller moment of inertia (*I*_min⁡_, Table 1), loss of IR in osteoblasts caused a decrease in the structural strength of the femur in bending, compared with IR flox bones (Figure 2: peak force). A decrease in cortical tissue mineral density (Ct.TMD, Table 1) was also seen in female OIRKO mice. However, there was no difference in the estimated bending strength between genotypes.

As determined by the ratio of bone length to total cross-sectional area (Tt.AR), the femur slenderness for OIRKO mice was considerably greater than the femur slenderness for IR flox bones. This structural impairment in the OIRKO mice was decoupled from body weight, because the ratio of slenderness to body weight (Length/Tt.AR per BW) was higher for knockout than for control mice of both genders (Figures 2(a) and 2(b)). That is, bones from OIRKO mice were more slender (i.e., more susceptible to failure) than expected for their reduced body weight.

While* Cre* expression alone in the* (Osx)-Cre* ^+/−^ male mouse has been shown in one study to negatively affect weight at 4 weeks of age, the effect of* Cre* was not evident by 12 weeks of age [28]. We also observed no differences in weight between* (Osx)-Cre* ^−/−^ and (*Osx)-Cre* ^+/−^ male mice at 8 weeks of age (Supplemental Table  1). Moreover, those skeletal parameters that* were* significantly affected in the OIRKO phenotype (Table 1)* were not* significantly different between* Cre* ^−/−^ and* Cre* ^+/−^ genotypes, suggesting that the skeletal phenotype observed in male and female OIRKO mice is accounted for principally through the diminished expression of the IR.

Because insulin signaling in bone has been linked by others to a feed-forward mechanism to activate osteocalcin, which in turn regulates glucose homeostasis [21, 22], we also examined the metabolic phenotype of the OIRKO mice (Table 2). In this model, there were no significant abnormalities in fasting blood glucose or insulin levels or in glucose or insulin dynamics after a glucose challenge (Table 2) in OIRKO mice compared with IR flox mice. Moreover, total osteocalcin levels were not significantly different between groups in either male or female mice. While undercarboxylated osteocalcin has been reported to be lower in mice in which the IR has been eliminated in mature osteoblasts [21, 22], this was not measured in our study due to the lack of a validated and commercially available assay for undercarboxylated osteocalcin in mice.

Osteoclast activity was assessed by examining systemic concentrations of c-terminal telopeptide of type 1 collagen (i.e., RatLAPS). C-terminal telopeptide of type 1 collagen was modestly lower in male OIRKO mice (Table 2), compared with IR flox mice, but not different when genders were combined (*P* = 0.15). Osteogenesis was further assessed by measuring systemic concentrations of PINP; these concentrations were not different between OIRKO mice and IR flox mice (*P* = 0.97).

## 4. Discussion

Elimination of the insulin receptor specifically in osteoprogenitor cells affords the opportunity to study the impact of the IR and its ligand* in vivo* on bone development, without the metabolic consequences associated with eliminating the IR in all tissues, which consistently leads to hyperinsulinemia. OIRKO mice also provide a model of osteoblast-specific decreased insulin action, as expected in patients with suboptimally controlled T1D, but without concurrent hyperglycemia. As such, loss of IR in the osteoblast lineage resulted in a growth-constricted phenotype. Although the femur length, indicative of appendicular bone growth, was reduced in the OIRKO, the thickness of the diaphyseal cortex was more greatly affected, resulting in a more slender femur which manifested a decrease in the structural strength. In male mice alone, decreased metaphyseal trabecular bone, with thinner and more rodlike trabeculae, was also observed. Moreover, the skeletal phenotype of the OIRKO mouse appears more severe than that reported using the* Col1a1-Cre *or the* OC-Cre* construct [21, 22], suggesting that earlier elimination of the IR in osteoblast development may provide further insights into its overall role in osteoblastogenesis.

The reduced body size of both male and female OIRKO mice was evident as early as at 4 weeks of age and persisted throughout this study. By 10–12 weeks of age, a microarchitectural skeletal phenotype was clearly evident. It remains to be seen how elimination of IR in this model will impact skeletal integrity, bone strength, and bone healing as OIRKO mice age further, though more profound deficits may become apparent in older mice. Studies are ongoing to address this question.

In mice made insulin deficient, insulin treatment can restore trabecular architecture, suggesting that insulin may be involved in trabecular homeostasis [19]. Fulzele et al. have shown that lack of insulin receptor signaling in osteoblasts alters trabecular acquisition as evidenced by decreased BV/TV, Tb.N, and Tb.Th and increased Tb.Sp in young male mice (3–6 weeks of age) lacking IR expression in mature osteoblasts [22]. However, these differences appear to dissipate as the mice age [22]. In the current study, BV/TV and Tb.Th were decreased in male OIRKO mice, consistent with the previous findings by Fulzele et al. [22]; however, we did not study older OIRKO mice to see if the trabecular phenotype persists. Indeed, in slightly older female OIRKO mice, no major differences were noted in the trabecular compartment; however, these differences between OIRKO male and female mice might not simply be attributable to age but may reflect the impact of gender, as estrogen has a protective effect on metaphyseal bone [29].

As in previous studies investigating osteoblast-specific deletion of IR in male mice [21, 22], we too observed a modest reduction in a serum marker of bone resorption in the male OIRKO mouse (but not in the female OIRKO mouse). Nevertheless, decreased resorption in male OIRKO mice did not translate into elevated glucose levels or changes in insulin dynamics in the OIRKO model. This lack of glucose or insulin dysregulation varies from the observations of others in male mice, wherein IR expression in osteoblasts was reduced using* OC-Cre* and* Col1a1-Cre* [21, 22]. It was anticipated that eliminating the IR in osteoprogenitors rather than in more mature osteoblasts might cause an even more profound effect on glucose homeostasis, more consistent with frank diabetes. However, within each gender of OIRKO mice, no significant differences in glucose tolerance were seen, suggesting a more complex connection between osteoblasts and energy metabolism than previously appreciated. Additionally, total osteocalcin levels were not significantly different between IR flox and OIRKO mice. Total osteocalcin levels have been reported to be suppressed in male mice in which the IR was eliminated from mature osteoblasts using the* OC-Cre* construct, yet when the IR in osteoblasts is eliminated using the* Col1a1-Cre, *no difference in osteocalcin levels was observed [21, 22], similarly to the OIRKO mouse.

At present, little is known about the specific mechanisms underlying diabetic bone disease. While the current study does not yet elucidate mechanistic pathways by which insulin signaling is critical to osteoblastogenesis, we can infer that insulin receptor expression in osteoblasts is quite important for proper bone development and maintenance of structural integrity. Further mechanistic analysis of the OIRKO genotype in future studies will be necessary to explain the observed phenotype, and studies using these genetically modified mice should broaden our understanding of diabetic bone disease. From the present studies, however, we hypothesize that, in T1D, insulin deficiency leading to long-term decreased insulin receptor signaling in the growing bones of children and adolescents with this disease might suppress periosteal bone expansion and alter the bone microarchitecture, increasing susceptibility to fracture in later years. Validating this hypothesis in humans also requires further investigation.

## Supplementary Material

To ascertain if any skeletal differences in the OIRKO mice could be attributed to the Cre transgene alone, selected properties of bone, as determined by µCT and three-point bending, were examined by comparing 8- week old Cre-/- and Cre+/- genotypes. Results are shown in Supplemental Table 1. Those skeletal parameters that were significantly affected in the OIRKO phenotype (Table 1: BV/TV, Tb.Th, SMI, Ma.V, Ct.Th, Imin, Ct.Ar, Slenderness, Ct.TMD, and Stiffness), were not significantly different between Cre-/- and Cre+/- genotypes. Body weight and femur length were also not different between Cre-/- and Cre+/- genotypes. This suggested that the skeletal phenotype of OIRKO mice was accounted for by diminished expression of insulin receptor in osteoblasts. (Abbreviations are defined in Table 1. Significant differences are highlighted in bold font.)

## Figures and Tables

**Table 1:** Selected properties of bone, as determined by *μ*CT and three-point bending, for males (top) and females (bottom), comparing control (IR flox) and OIRKO mice.

Property (male)	Units	IR flox (*n* = 6)	OIRKO (*n* = 9)	*P*-value
Metaphysis (trabecular bone)				
BV/TV	%	11.71 ± 0.94	8.95 ± 2.04	**0.009**
Tb.N	mm^−1^	5.15 ± 0.33	4.95 ± 0.61	0.47
Tb.Th	mm	0.0392 ± 0.002	0.0344 ± 0.002	**<0.001**
Tb.Sp	mm	0.195 ± 0.013	0.206 ± 0.028	0.38
SMI^a^	—	2.33 ± 0.08	2.61 ± 0.20	**0.006**
Tb.TMD	mgHA/cm^3^	901 ± 8	888 ± 17	0.10

Diaphysis (cortical bone)				
Ma.V	mm^3^	1.22 ± 0.12	1.03 ± 0.11	**0.005**
Ct.Th	mm	0.148 ± 0.011	0.124 ± 0.011	**0.001**
Imin	mm^4^	0.102 ± 0.007	0.066 ± 0.009	**<0.001**
Ct.Ar	mm^2^	0.640 ± 0.033	0.4892 ± 0.044	**<0.001**
Length	mm	13.70 ± 0.35	12.59 ± 0.36	**<0.001**
Slenderness^b^	mm/mm^2^	20.8 ± 0.7	25.1 ± 2.2	**<0.001**
Ct.TMD	mgHA/cm^3^	1185 ± 27	1176 ± 25	0.49
Stiffness	N/mm	40.7 ± 8.5	36.9 ± 12.6	0.57
Modulus	GPa	4.2 ± 0.8	6.1 ± 2.6	0.137
Bending strength	MPa	147 ± 17	172 ± 36	0.177

Property (female)	Units	IR flox (*n* = 6)	OIRKO (*n* = 9)	*P*-value

Metaphysis (trabecular bone)				
BV/TV	%	4.38 ± 0.92	4.84 ± 1.52	0.52
Tb.N	mm^−1^	3.37 ± 0.33	3.35 ± 0.59	0.95
Tb.Th	mm	0.037 ± 0.002	0.036 ± 0.004	0.53
Tb.Sp	mm	0.302 ± 0.032	0.316 ± 0.084	0.69
SMI^a^	—	3.19 ± 0.17	2.80 ± 0.18	**0.001**
Tb.TMD	mgHA/cm^3^	916 ± 13	901 ± 30	0.28

Diaphysis (cortical bone)				
Ma.V	mm^3^	1.07 ± 0.07	1.01 ± 0.08	0.16
Ct.Th	mm	0.162 ± 0.005	0.138 ± 0.011	**<0.001**
Imin	mm^4^	0.093 ± 0.010	0.072 ± 0.007	**<0.001**
Ct.Ar	mm^2^	0.651 ± 0.028	0.538 ± 0.048	**<0.001**
Length	mm	14.41 ± 0.39	13.22 ± 0.45	**<0.001**
Slenderness^b^	mm/mm^2^	21.3 ± 0.687	23.5 ± 1.236	**0.002**
Ct.TMD	mgHA/cm^3^	1251 ± 15	1209 ± 13	**<0.001**
Stiffness	N/mm	80.6 ± 11.5	57.3 ± 6.7	**<0.001 **
Modulus	GPa	9.4 ± 1.5	8.5 ± 1.2	0.26
Bending strength	MPa	166 ± 10	161 ± 13	0.50

^a^Structural model index characterizes the shape of trabecular bone (1: platelike; 3: rodlike). ^b^Slenderness is the ratio of the length to the total cross-sectional area of the femur midshaft. Abbreviations: BV/TV: bone volume/tissue volume; Tb.N: trabecular number; Tb.Th: trabecular thickness; Tb.Sp: trabecular separation; SMI: structural model index; Tb.TMD: trabecular tissue mineral density; Ma.V: marrow volume; Ct.Th.: cortical thickness; Imin: moment of inertia; Ct.Ar: cortical area; Ct.TMD: cortical tissue mineral density. Significant differences are highlighted in bold font.

**Table 2:** Metabolic phenotype of males (top) and females (bottom), comparing control (IR flox) and OIRKO mice.

	Units	IR flox (*n* = 6)	OIRKO (*n* = 9)	*P* value
Measurements (male)				
Final body weight	g	24.7 ± 2.2	19.3 ± 2.0	**<0.001**
Fasting insulin	ng/mL	0.14 ± 0.04	0.26 ± 0.22	0.24
Fasting blood glucose	mg/dL	67.8 ± 11.4	68.11 ± 11.4	0.97
Insulin AUC	—	43.8 ± 17.0	40.5 ± 8.9	0.63
Glucose AUC	—	46,660 ± 10,660	43,450 ± 10,328	0.57
OC	ng/mL	109.6 ± 9.7	99.5 ± 38.7	0.54
RatLAPS	ng/mL	25.1 ± 2.3	21.3 ± 3.8	**0.05 **

Measurements (female)				
Final body weight	g	23.1 ± 2.6	17.6 ± 2.4	**0.001**
Fasting blood glucose	mg/dL	52.7 ± 7.6	52.6 ± 11.6	0.98
Glucose AUC	—	27,781 ± 3985	30,695 ± 5300	0.27
OC	ng/mL	120.7 ± 38.1	92.5 ± 35.0	0.16
RatLAPS	ng/mL	25.5 ± 5.1	23.8 ± 7.6^a^	0.64

Abbreviations: AUC: Area under the curve; OC: osteocalcin; RatLAPS; a marker of bone resorption. ^a^ *n* = 8 for this measurement. Significant differences are highlighted in bold font.
