# Supplementary material for: Loss of Insulin Receptor in Osteoprogenitor Cells Impairs Structural Strength of Bone
Source: J Diabetes Res. 2014 May 18;2014:703589. doi: 10.1155/2014/703589 (PMC4052184; doi:10.1155/2014/703589)
Supplement: Supplementary file 1 — To ascertain if any skeletal differences in the OIRKO mice could be attributed to the Cre transgene alone, selected properties of bone, as determined by µCT and three-point bending, were examined by comparing 8- week old Cre-/- and Cre+/- genotypes. Results are shown in Supplemental Table 1. Those skeletal parameters that were significantly affected in the OIRKO phenotype (Table 1: BV/TV, Tb.Th, SMI, Ma.V, Ct.Th, Imin, Ct.Ar, Slenderness, Ct.TMD, and Stiffness), were not significantly different between Cre-/- and Cre+/- genotypes. Body weight and femur length were also not different between Cre-/- and Cre+/- genotypes. This suggested that the skeletal phenotype of OIRKO mice was accounted for by diminished expression of insulin receptor in osteoblasts. (Abbreviations are defined in Table 1. Significant differences are highlighted in bold font.) [file 703589.f1.pdf]

SUPPLEMENTAL TABLE 1

PROPERTY	Units	$Cre^{-/-}$ (n=18)	$Cre^{+/-}$ (n=9)	p-value
Body weight	grams	24.36 ± 2.61	23.83± 2.09	0.60
**Metaphysis (Trabecular bone)**				
BV/TV	%	16.5 ± 4.4	14.9 ± 4.0	0.40
Tb.N	mm-1	5.6 ± 0.61	5.67 ± 0.65	0.80
Tb.Th	mm	0.046 ± 0.006	0.044 ± 0.007	0.36
Tb.Sp	mm	0.179 ± 0.023	0.177 ± 0.02	0.83
SMIa	--	2.032 ± 0.373	2.218 ± 0.299	0.21
Tb.TMD	mgHA/cm3	893 ± 10.6	880 ± 19.5	**0.02**
**Diaphysis (Cortical bone)**				
Ma.V	mm3	1.27 ± 0.158	1.36 ± 0.209	0.22
Ct.Th	mm	0.165 ± 0.017	0.156 ± 0.013	0.18
Imin	mm4	0.115 ± 0.027	0.118 ± 0.028	0.79
Ct.Ar	mm2	0.717 ± 0.098	0.693 ± 0.084	0.53
Length	mm	13.742 ± 0.622	13.71 ± 0.655	0.90
Slendernessb	mm/mm2	19.457 ± 2.47	19.999 ± 2.08	0.58
Ct.TMD	mgHA/cm3	1153.6 ± 25.7	1133.6 ± 23.7	0.06
Stiffness	N/mm	75.75 ± 16.1	70.59 ± 17.23	0.45
Modulus	GPa	7.21 ± 1.71	6.5 ± 1.3	0.28
Bending strength	MPa	165.15 ± 19.36	167.7 ± 16.23	0.74
Peak Force	N	14.76 ± 2.5	14.93 ± 2.91	0.88
